# Editorial Notes

**Published:** 1892-01

**Authors:** 


### Editorial.

CHRISTMAS has died and been buried, and from out the sepulcher the New Year comes forth smiling and happy, and full of gracious promises, and to all our readers and friends we extend our best wishes for their continued happiness and prosperity, and the wish that each returning New Year break like a radiant ray of sunlight into your lives, making for each of you a year, each page of which is illuminated with characters of love and enjoyment and wishes fulfilled.

---

SOME changes occur in our editorial staff with the New Year, Drs. Stockton and Nicolson retiring. The former returns to his old home, Newark, N. J., to resume the practice of his specialty with his preceptor.

The SOUTHERN MEDICAL RECORD also changes its cover, and with the hearty co-operation of our friends, we hope to improve the excellent standard this journal has already established, and by their hearty co-operation, we do not mean your subscription alone, but your contributions to the literary department as well.

---

THERE are no class of practitioners better able to report interesting cases and experiences than the country doctor. Isolated as they so frequently are, miles from consultation or assistance where all classes of work come under their observation and treatment, they are necessarily self-reliant, depending as they have to do on their own wit and shrewdness for diagnosis and treatment, and their ingenuity to improvise and supply the place of apparatus that may be necessary in special cases. And it is a very rare occurrence when they are not able to meet successfully all emergencies.

---

THERE is only one thing that these men are laggards about, only one complaint that we make against them, and it is that they will not write out and report their cases. Whenever you meet them, they tell you of any number of interesting cases and experiences; even promise to write them out for you, but that is the last ever heard of the case.

The amount of material piled away in their brains that would prove of infinite value if contributd to the literature of medicine and surgery is astonishing, for in nearly every radical change in either branch they have been the pioneers, and they have been to the profession what the pioneer has always been—the advance guard of civilization.

---

WE ASK these men to send us contributions of these cases and experiences. They not only owe it to themselves but to the profession. How often it occurs to a physician to see an idea published to the world, a thought which long before he had dismissed from his notice, as without value because it was his. Abide by and publish your impressions and convictions, or else tomorrow a stranger will say with masterly good grace what you have thought all the time, and you will have to receive with shame your own opinion from another. Have the courage of your convictions to publish and promulgate them, to take up the gauntlet in their defense—develop an idea with the patience exhibited by Marion Sims, who operated over thirty times on his patient before he met with complete success. The anticipations of yesterday defeated by the realizations of today; but each time recognizing and correcting an error, standing by his conviction, though his professional friends refused any longer to assist him. He remembered that time does not preserve that which has cost no time to create. How time has vindicated him and what a monument suffering womanhood should erect to his memory!

---

IT IS more than passing strange the mistakes made by prominent medical men as to whom honors are due for valuable discoveries contributed to medicine. For instance, a physician told me he heard Mr. Lawson Tait make the assertion in a medical address, delivered to a British Association, that the greatest achievement, the greatest progress in surgery was the discovery of anæsthesia by Dr. James Y. Simpson. Why, any first course student should know better; we advise him to study the literature of the subject. His investigation will show him that anæsthesia was first used in England by Mr. Robinson, a London dentist, on December 19, 1846, and a hospital patient was operated on under anæsthesia by the celebrated surgeon, Mr. Liston, on December 21st, the same year Dr. James Y. Simpson first used it to relieve the pains of child-birth on Jan. 19, 1847—just a month after its first use in England.

---

WE are glad to welcome to our midst Dr. W. E. B. Davis, who moves from Birmingham, Ala., to become associated with Dr. J. B. S. Holmes, of Rome, Ga. Dr. Davis is President of the Tri-State Medical Association, and as Secretary of the Southern Surgical and Gynæcological Association, he has contributed more to its success than any one. The Doctor has contributed some valuable papers to the literature of gynæcology and abdominal surgery, and will prove himself a valuable accession to the profession of this State. W. F. W.

